# Hospital Transfer Between Primary and Secondary Metabolic Bariatric Surgery in The Netherlands: A Cross-sectional Multi-party Computation Analysis of Frequency and Associated Factors

**DOI:** 10.1007/s11695-025-08284-8

**Published:** 2025-10-20

**Authors:** Floris F. E. Bruinsma, Steven van Schuppen, Ronald S. L. Liem, Perla J. Marang-van de Mheen, Simon W. Nienhuijs

**Affiliations:** 1 Maastricht University Medical Centre, Maastricht, Netherlands; 2 Scientific Bureau, Dutch Institute for Clinical Auditing, Leiden, Netherlands; 3 Groene Hart Hospital, Gouda, Netherlands; 4 Nederlandse Obesitas Kliniek, The Hague and Gouda, Netherlands; 5 Delft University of Technology, Delft, Netherlands; 6 Catharina Hospital, Eindhoven, Netherlands

**Keywords:** Secondary metabolic bariatric surgery, Revision surgery, Revisional surgery, Multi-party computation, Secure multi-party computation, Hospital transfer, Hospital switching, Second opinion

## Abstract

**Introduction:**

Some patients undergoing metabolic bariatric surgery (MBS) may transfer to another hospital for subsequent procedures. Due to legal constraints imposed by privacy regulations on inter-hospital data sharing, limited research has examined the characteristics and outcomes of these patients. This study aimed to identify the frequency and factors associated with hospital transfer using a novel privacy-enhancing approach based on secure multi-party computation (MPC).

**Methods:**

All primary and secondary MBS procedures registered in the Dutch Audit for Treatment of Obesity between January 1, 2014, and December 31, 2022, were considered. MPC enabled privacy-preserving linkage of surgeries across different hospitals. Patients undergoing secondary surgery in the same or a different hospital were compared on patient and treatment characteristics and outcomes of primary MBS to investigate associations with hospital transfer.

**Results:**

Two thousand three hundred eighty-two patients with data on both primary and secondary MBS were identified. A minority (*n* = 275; 11.5%) underwent their second procedure elsewhere. At baseline, these patients on average were younger (37.9 vs. 42.5, *p* < 0.001), less often had hypertension or GERD, and had similar BMI (43.9 vs 43.9, *p* = 0.89) compared with those who stayed. At secondary surgery, the BMI of patients transferring hospitals on average was lower (39.0 vs. 43.0, *p* < 0.001), and the indication was more often recurrent weight gain (49.0% vs. 23.0%, *p* < 0.001).

**Conclusion:**

A minority of patients (1 in 9) transferred to a different hospital for secondary MBS. These patients were generally younger and had fewer obesity-related diseases. Although they presented with a lower BMI at the time of secondary surgery, they more frequently sought surgery for recurrent weight gain.

**Supplementary Information:**

The online version contains supplementary material available at 10.1007/s11695-025-08284-8.

## Introduction

The number of patients undergoing metabolic bariatric surgery (MBS) has been increasing for many years [1, 2]. As obesity is a chronic and relapsing disease [3], patients may require secondary MBS due to unsatisfactory weight loss or complications from the initial procedure. Although MBS remains the most effective obesity treatment [4, 5], secondary surgery is becoming increasingly common, already accounting for 10–20% of cases in many countries [2]. While primary MBS is becoming more balanced in terms of finding the optimal treatment strategy, secondary procedures often yield less favorable results, and a significant variability in surgical approaches exists [6]. This highlights the need for focused research on secondary MBS.

Registry data, such as that from the Dutch Audit for Treatment of Obesity (DATO), can provide valuable insights into outcomes of secondary MBS. However, meaningful interpretation requires linkage with data from the primary procedure to put the outcomes of the secondary surgery in context, as both surgeries form part of the patient’s treatment. Such linkage is straightforward when both operations are performed in the same hospital, but becomes problematic when they occur at different institutions. Privacy regulations, imposed by the European General Data Protection Regulation (GDPR) [7], prohibit cross-institutional linkage, restricting analyses in DATO and other quality registries to patients treated within a single hospital. To assess the validity and representativeness of such analyses, it is essential to determine how often patients transfer to another hospital for secondary MBS and whether these patients differ systematically from those who remain at the same institution.

**Fig. 1:**
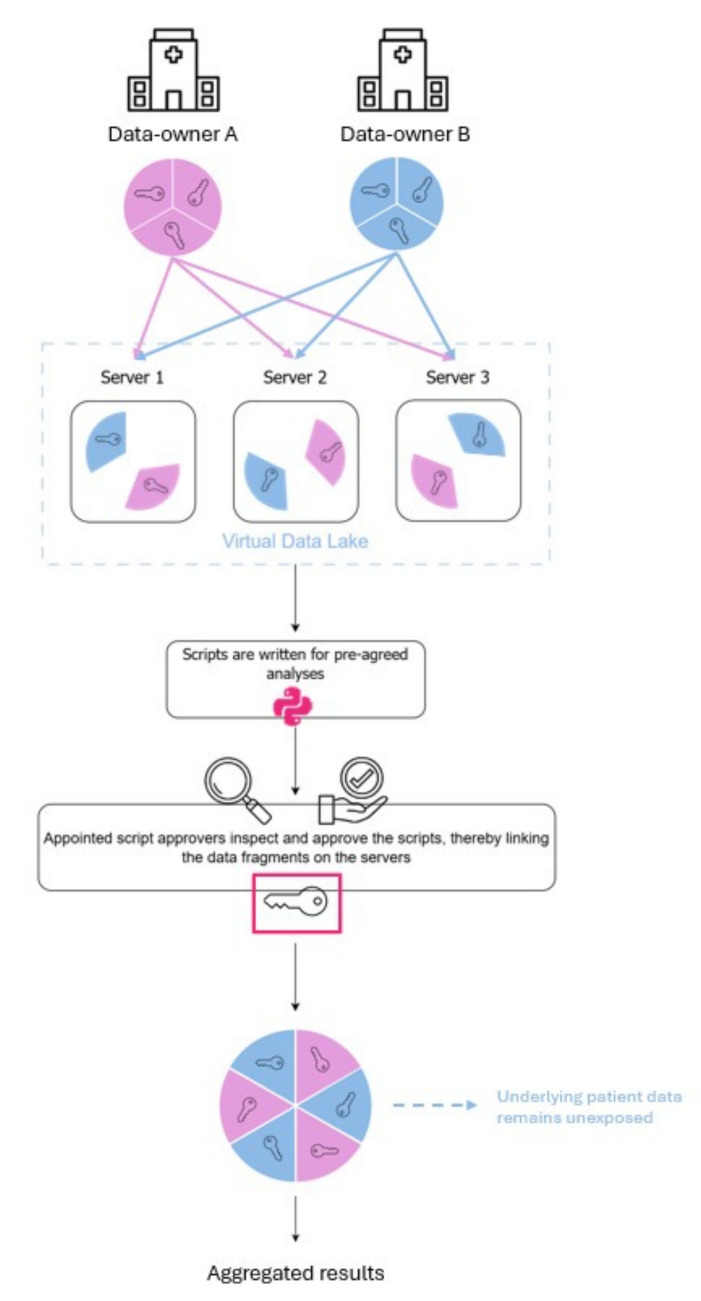
Visualization of the secure multi-party computation process

Addressing this limitation requires novel strategies for secure data linkage across institutions. A promising approach is secure multi-party computation (MPC), which enables collaborative analysis of sensitive information while preserving patient privacy. MPC relies on locally encrypting, fragmenting, and distributing the data across multiple independent servers hosted by a trusted third party (see Fig. 1), allowing analyses on the collectively processed data without exposing individual-level information to researchers or participating hospitals [8, 9]. Thereby, MPC facilitates the secure coupling of primary and secondary MBS data across different hospitals.

Therefore, the aim of this study was to evaluate the frequency of hospital transfers for secondary MBS in the Netherlands using MPC and identify whether patients who transfer hospitals differ in patient profiles or the outcomes of their primary surgery compared with patients who remain in the same hospital for secondary MBS. Understanding these factors is critical for interpreting results of future research on secondary MBS and for improving the management of secondary MBS care.

## Methods

### Setting

Data was obtained from the Dutch Audit for Treatment of Obesity (DATO), the mandatory MBS registry from the Netherlands [10, 11]. DATO collects data on patients, procedures, and outcomes, and previous data verification has shown high data validity [12]. All patients undergoing primary or secondary MBS from 2014 onwards are included in this database. As DATO is an opt-out registry, informed consent was not required under Dutch law.

### Secure Multi-party Computation

Dutch regulations, in compliance with European legislation (i.e., GDPR) [7], prohibit the exchange of patient-sensitive information (e.g., social security numbers) for medical research. As a result, registry data from the same patient across different hospitals cannot be connected in DATO. MPC presents a potential solution, as it enables patient linkage without exchanging patient-sensitive data [8, 9]. The MPC software used in this study was provided by Roseman Labs [13]. MPC relies on locally encrypting the data and dividing the encrypted variables into multiple parts, which are then uploaded to multiple servers of a trusted third party. With an authorization key only available to the researchers, analyses can be performed on this interlinked data without ever exposing the underlying (patient-sensitive) data to anyone (Fig. 1). This way, hospital data is available for analysis but not exchanged between hospitals. In DATO, each hospital assigns its own patient identifiers, which enable linkage within the same hospital but prevent direct linkage of surgeries when patients transfer between hospitals. To enable such linkage, alternative patient identifiers (APIs) were developed based on combinations of family name, date of birth, biological sex, and name particles, which were encrypted and matched using MPC. The combination of family name, date of birth, and biological sex resulted in the most reliable patient matches. Surgeries that could be matched by the API but not by the original identifier were classified as from patients transferring hospitals. For more details on the data coupling process, see the supplemental materials.

### Patients

All primary and secondary MBS procedures registered in DATO between January 1, 2014, and December 31, 2022, were uploaded to the MPC framework. Patients were categorized based on whether they underwent their secondary procedure in the same hospital or transferred to a different hospital for the intervention, and these groups were compared. Only procedures that alter the gastrointestinal anatomy are considered secondary MBS, such as the creation of a new anastomosis or the resection of part of the gastrointestinal tract. Secondary MBS can be further categorized into three subtypes: revisional surgery, which entails modifications within the same surgical technique (e.g., limb length adjustment within RYGB); conversional surgery, which involves changing from one type of MBS to another (e.g., SG to RYGB); and undo surgery, which refers to the complete reversal of a previous MBS procedure, restoring normal gastrointestinal anatomy. Procedures aimed solely at correcting complications without altering gastrointestinal anatomy, such as the repair of internal herniation, were not classified as secondary MBS.

### Outcomes and Statistical Analysis

The frequency of hospital transfers among patients receiving both primary and secondary MBS in the specified period was determined. Characteristics of patients who transferred and those who did not were compared on baseline age, sex, body mass index (BMI), American Society for Anesthesiologists (ASA) score, and obesity-related diseases (i.e., diabetes mellitus, hypertension, dyslipidemia, obstructive sleep apnea syndrome (OSAS), gastro-esophageal reflux disease (GERD), and musculoskeletal pain). Differences in age and BMI at the time of secondary surgery were also assessed. These characteristics, along with indications for secondary surgery and the time to secondary MBS, were compared between patients who transferred hospitals and those who did not. Additionally, the occurrence of severe complications (i.e., Clavien-Dindo [14, 15] grade 3b or higher) after the primary surgery was assessed to explore whether this was related to hospital transfers. Differences in categorical variables were analyzed using chi-square tests, and normally distributed variables were assessed using independent samples *t*-tests. Non-normally distributed variables were analyzed using the Kruskal-Wallis tests. Analyses were performed in Python using a Pandas derivative package specially created for conducting analyses on MPC data (Crandas package) [16, 17].

### Sensitivity Analysis

Some secondary MBS procedures may occur shortly after the primary MBS procedure due to complications, such as gastrointestinal leakage needing revision of an anastomosis or anatomical obstructions (e.g., stenotic anastomosis or kinking of the entero-enterostomy). These patients are unlikely to choose another hospital during this period as they are fully integrated into the local care process and receive ongoing postoperative care. Therefore, a sensitivity analysis was conducted, excluding patients who underwent secondary surgery within 90 days of the primary procedure to ensure that their initial postoperative treatment period was completed, thereby equalizing the opportunity for all patients to consider hospital transfer.

## Results

**Fig. 2:**
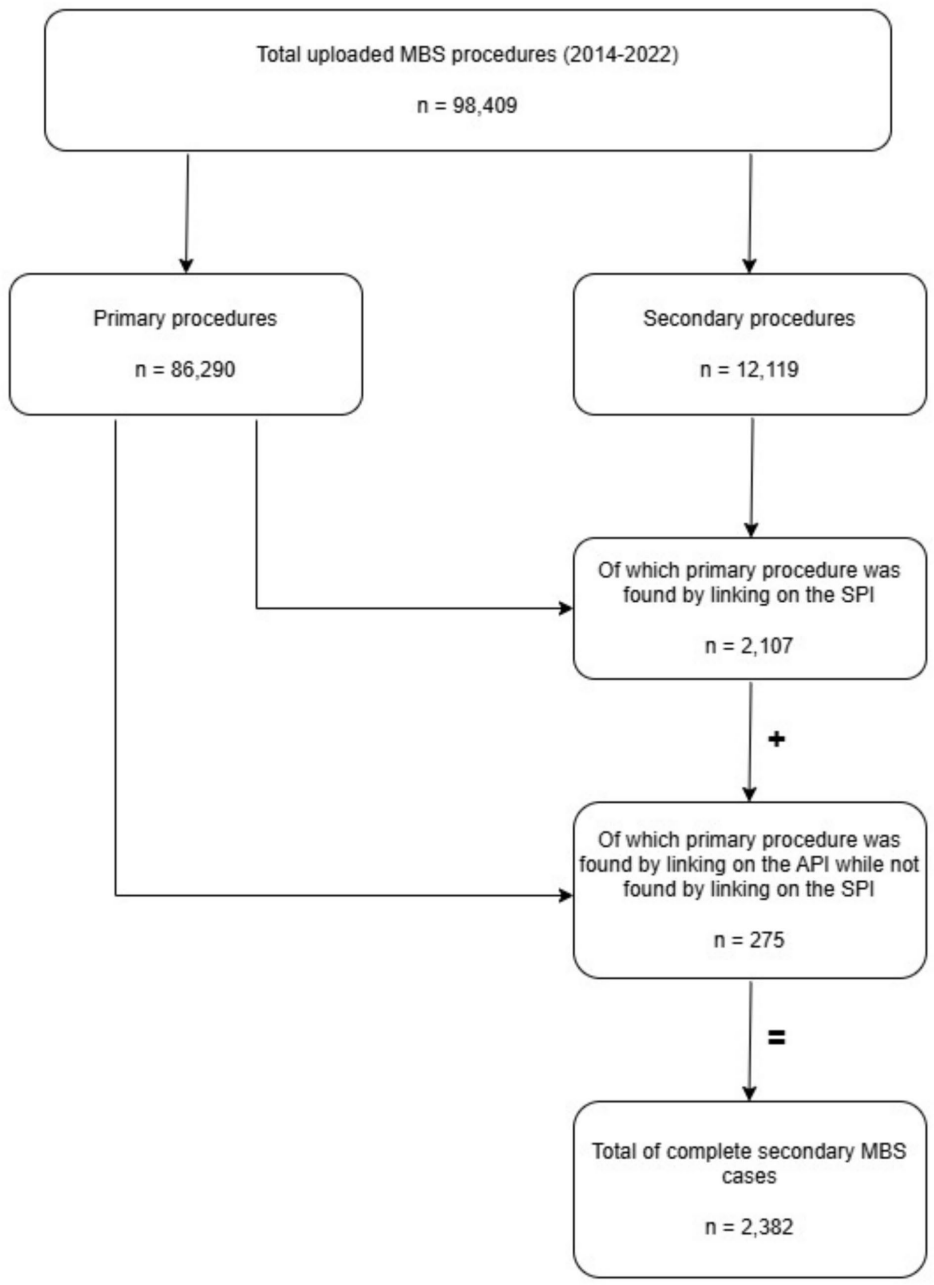
Patient inclusion flow diagram. MBS metabolic bariatric surgery, SPI standard patient identifier, API alternative patient identifier

In total, 98,409 encrypted surgical records were searched, and 2382 (2.4%) patients who received both primary and secondary MBS in the specified period were identified (see Fig. 2). Among these patients, 275 (11.5%) transferred to another hospital for the secondary procedure, while 2107 (88.5%) received the secondary surgery in the same hospital. Patients who transferred to another hospital had similar BMIs at primary surgery (43.9 vs 43.9, difference = 0.1, 95% confidence interval (CI) −0.8 to 0.9, *p* = 0.89), were younger (37.9 vs. 42.5, difference = 4.6, 95% CI 3.1–6.1, *p* < 0.001), and less often had hypertension or GERD (Table 1). They more frequently received a gastric bypass, while those staying in the same hospital more often had received sleeve gastrectomy (SG) as the primary procedure. Patients transferring hospitals more frequently received primary Roux-en-Y gastric bypass (RYGB) and less often one anastomosis gastric bypass (OAGB) (Table 1). The mean (SD) time between the two procedures was 4.1 (2.3) years for patients who transferred and 2.8 (2.1) years for those who stayed. At the time of secondary surgery, patients transferring to a different hospital were still younger, although the difference was smaller (42.0 vs. 45.4, difference = 3.4, 95% CI 1.9–4.8, *p* < 0.001), and had lower BMI (39.0 vs. 43.0, difference = 4.1, 95% CI 3.1–5.0, *p* < 0.001) (Table 2). The type of secondary surgery was mainly conversion surgery in both groups, but patients transferring hospitals more frequently underwent revision surgery (36.7% vs. 24.1%, difference = 12.6, 95% CI 0.6–24.6, *p* < 0.05). Among patients who transferred between hospitals, secondary surgery was more frequently performed for recurrent weight gain (RWG) (49.0% vs 23.0%, difference = 26.0, 95% CI 17.7–34.3, *p* < 0.001) and less often for GERD (4.8% vs 10.2%, difference = 5.4, 95% CI 1.6–9.2, *p* < 0.05). Patients who transferred hospitals less frequently experienced severe postoperative complications after the primary surgery (1.1% vs. 3.6%, difference = 2.5, 95% CI 1.0–4.0, *p* < 0.05).

**Table 1:** Patient and procedure characteristics at the time of the primary surgery

		Same hospital	Hospital transfer	*p*-value	*N*
*N*		2107	275		
Age at baseline, mean (SD)		42.5 (11.8)	37.9 (11.6)	< 0.001	2382
Sex, *n* (%)	Female	1751 (83.1)	229 (83.3)	0.94	2382
BMI at baseline, mean (SD)		43.9 (6.9)	43.9 (6.0)	0.89	2059
ASA score, *n* (%)	< 3	906 (43.7)	163 (60.1)	< 0.001	2346
	3+	1169 (56.3)	108 (39.9)		
Diabetes mellitus, *n* (%)	Not present	1449 (80.7)	217 (82.8)	0.49	2058
	Present without medication	124 (6.9)	13 (5.0)		
	Present with medication	223 (12.4)	32 (12.2)		
Hypertension, *n* (%)	Not present	1181 (65.8)	192 (73.3)	< 0.05	2058
	Present without medication	234 (13.0)	29 (11.1)		
	Present with medication	381 (21.2)	41 (15.6)		
Dyslipidemia, *n* (%)	Not present	1462 (81.4)	223 (85.1)	0.32	2058
	Present without medication	168 (9.4)	21 (8.0)		
	Present with medication	166 (9.2)	18 (6.9)		
OSAS, *n* (%)	Not present	1483 (82.6)	219 (83.6)	0.52	2058
	Present without medication	159 (8.9)	18 (6.9)		
	Present with medication	154 (8.6)	25 (9.5)		
GERD, *n* (%)	Not present	1407 (78.6)	224 (85.8)	< 0.05	2051
	Present without medication	211 (11.8)	21 (8.0)		
	Present with medication	172 (9.6)	16 (6.1)		
Musculoskeletal pain, *n* (%)	Not present	939 (52.3)	157 (59.9)	0.06	2056
	Present without medication	807 (45.0)	101 (38.6)		
	Present with medication	48 (2.7)	4 (1.5)		
Type of primary surgery, *n* (%)	LAGB	54 (2.6)	22 (8.1)	< 0.001	2382
	SG	1196 (58.2)	120 (44.0)		
	Bypass	804 (39.1)	131 (48.0)		
	Other	53 (2.5)	2 (0.7)		
Type of primary bypass*, *n* (%)	RYGB	456 (56.9)	120 (91.6)	< 0.001	933
	OAGB	246 (30.7)	7 (5.3)		
	Ring augmented RYGB	100 (12.5)	4 (3.1)		
	Missing**	2 (0.2)	0 (0.0)		

*N* number of patients, *SD* standard deviation, *BMI* body mass index, *ASA* American Society of Anesthesiologists, *OSAS* obstructive sleep apnea syndrome, *GERD* gastro-esophageal reflux disease, *LAGB* laparoscopic adjustable gastric banding, *SG* sleeve gastrectomy, *RYGB* Roux-en-Y gastric bypass, *OAGB* one anastomosis gastric bypass, * = only determined for patients receiving gastric bypass, ** = determined on the total number of patients who potentially could have had the variable filled (numbers do not add up to 100%)

**Table 2:** Patient and procedure characteristics of the secondary metabolic bariatric surgery

		Same hospital	Hospital transfer	*p*-value*
*N*		2107	275	
Age at revision, mean (SD)		45.4 (11.8)	42.0 (11.6)	< 0.001
BMI at revision, mean (SD)		43.0 (7.2)	39.0 (7.2)	< 0.001
Type of secondary surgery, *n* (%)	LAGB	46 (2.2)	13 (4.7)	< 0.001
	SG	52 (2.5)	22 (8.0)	
	Bypass	1485 (70.5)	200 (72.7)	
	Other	524 (24.9)	40 (14.5)	
Type of bypass**, *n* (%)	RYGB	1242 (83.9)	159 (79.5)	0.22
	OAGB	167 (11.3)	31 (15.5)	
	Ring augmented RYGB	72 (4.9)	10 (5.0)	
	Missing****	4 (0.3)	0 (0.0)	
Type of intervention, *n* (%)	Conversion	1202 (73.7)	86 (58.5)	< 0.001
	Undo	29 (1.8)	7 (4.8)	
	Revision	399 (24.5)	54 (36.7)	
	Missing****	477 (22.6)	128 (46.5)	
Type of revision***, *n* (%)	Gastro-enterostomy	94 (26.5)	10 (19.2)	0.12
	Entero-enterostomy	29 (8.2)	5 (9.6)	
	Adjustment of limb lengths	62 (17.5)	16 (30.8)	
	Other	170 (47.9)	21 (40.4)	
	Missing****	44 (11.0)	2 (3.7)	
Reason for intervention, *n* (%)	Primary non-responder	42 (2.6)	4 (2.7)	< 0.001
	Recurrent weight gain	376 (23.0)	72 (49.0)	
	Comorbidity progression	176 (10.8)	12 (8.2)	
	Excessive weight loss	52 (3.2)	3 (2.0)	
	GERD	166 (10.2)	7 (4.8)	
	Other	821 (50.3)	49 (33.3)	
	Missing****	474 (22.5)	128 (46.5)	
Years until secondary procedure, mean (SD)		2.8 (2.1)	4.1 (2.3)	< 0.001

*N* number of patients, *SD* standard deviation, *BMI* body mass index, *LAGB* laparoscopic adjustable gastric banding, *SG* sleeve gastrectomy, *RYGB* Roux-en-Y gastric bypass, *OAGB* one anastomosis gastric bypass, *GERD* gastro-esophageal reflux disease, * = missing category excluded from analyses, ** = only determined for patients receiving gastric bypass, *** = only determined for patients receiving revisional surgery, **** = determined on the total number of patients who potentially could have had the variable filled (numbers do not add up to 100%)

### Sensitivity Analysis

After excluding patients who received secondary surgery within the first 90 days, 2218 patients were included in the sensitivity analysis, of which 261 (11.8%) transferred to a different hospital. The mean (SD) time between both procedures was 4.4 (2.3) years for patients who transferred and 3.0 (2.1) years for those who stayed. The sensitivity analysis results were comparable to the primary analysis, with patients in the group transferring hospitals being younger and healthier (Supplementary Table 1). The types of surgeries performed and indications for secondary surgery remained consistent in both groups (Supplementary Table 2).

## Discussion

Using secure multi-party computation with real-world patient data, the proportion and characteristics of Dutch patients who transferred hospitals for secondary MBS and those who did not could be evaluated. Of all patients receiving both primary and secondary MBS between 2014 and 2022, one in nine transferred to another hospital for their secondary procedure. These patients were younger, had fewer obesity-related diseases, fewer severe postoperative complications, and more often received their secondary procedure for recurrent weight gain. The combination of a lower BMI at secondary surgery and a higher prevalence of recurrent weight gain as the indication may suggest that transferring patients are more easily dissatisfied with treatment outcomes, potentially leading them to seek second opinions; however, this requires further investigation.

While patient-initiated changes in healthcare providers are well-documented in various other medical fields [18–20], prior MBS research has not studied this phenomenon. Reasons for hospital transfers in other fields may be to receive more specialized care, for convenience, e.g., moving to another city, or just general dissatisfaction with the care received [21–23], suggesting that these factors may also influence MBS care decisions. Patients who transferred for secondary MBS were generally younger and had fewer obesity-related diseases, which may reflect age-related differences in healthcare behavior. A systematic review assessing second opinion patterns across 33 studies—albeit outside the MBS field—found that middle-aged patients, women, and those with higher education levels were more likely to seek a new care provider [24]. This aligns with a recent US survey of 20,000 patients, which reported that younger individuals were nearly six times more likely than older adults to change providers, primarily due to expectations of better care based on either personal experience or recommendations from others [25]. Only a minority (8%) cited logistical convenience, such as a more practical geographical location, as the reason for transferal. Those findings support the hypothesis that younger patients might be more critical or discerning regarding treatment outcomes. This is consistent with the current results, where transferring patients, despite having lower BMI at the time of secondary surgery, more often received secondary surgery for unsatisfactory weight loss. To assess the generalizability of these findings, similar analyses can be conducted in other countries to examine potential differences in patient and treatment characteristics associated with hospital transfer for secondary MBS.

Another insight from the results is that patients who stayed in their hospital more often had hypertension and GERD, potentially suggesting that patients might stay due to concurrent care provided by other specialists. The fact that the indication for secondary surgery was more often GERD in patients who stayed seems consistent with this explanation. Preoperative counselling on GERD after SG or OAGB, including the potential need for a conversional procedure to pursue resolution [26–29], may reduce patient dissatisfaction and the likelihood of seeking treatment elsewhere. The current results support this theory, as patients who stayed were more likely to have received primary SG, and when receiving gastric bypass, more often underwent OAGB. This may be explained by the limited options for revision after RYGB, which are associated with either suboptimal additional weight loss (e.g., pouch resizing, limb length distalization, or ring augmentation) [30–33] or high metabolic and nutritional complication rates (e.g., intestinal limb length alteration) [6, 34]. Meanwhile, SG can be converted relatively easily to RYGB, OAGB, or single anastomosis duodenal-ileal bypass with sleeve (SADI-S). Therefore, surgeons may be reluctant to offer secondary surgery for suboptimal weight loss after RYGB, leading to patients seeking a second opinion elsewhere [25]. Additionally, maintaining patient satisfaction requires preoperative counselling that addresses not only potential complications but also realistic expectations for weight loss. Because %TWL follows a normal distribution [35, 36], a proportion of patients will likely experience suboptimal outcomes. Patients should be made aware that they may fall at the lower end of this spectrum to prepare them for potentially unsatisfactory outcomes, rather than such results leading to dissatisfaction with their care.

Many countries have laws that protect personal health information to prevent misuse and encourage patients to be fully transparent during their consultation with healthcare professionals, ultimately benefiting patient care [7, 37, 38]. However, this legislation also hinders patient-data exchange for research that aims to improve patient care [39], and there is a need for pragmatic solutions. To our knowledge, MPC has been used with medical data from different data owners twice before [40, 41]. However, these studies were primarily designed as proof-of-principle experiments aimed at the technical aspects of MPC rather than applying the methodology to gain new insights. Additionally, only 192 and 48 patients were shared in those studies, resulting in relatively low computational demands. In contrast, nearly 100,000 surgical records were analyzed in the current study, thereby providing a robust test of the method’s capabilities. Given legal restrictions on sharing patient data between caregivers, MPC offers a solution for data analysis from multiple sources without requiring data exchange. Several well-established initiatives, such as EHDEN [42] and various Coordinated Registry Networks [43], are currently operational to facilitate data pooling across different institutions and countries. However, these projects do not aim to link shattered data from one patient but rather enable analyses on merged datasets to obtain new insights from larger data volumes. The current study serves as an example for other countries and study groups where researchers require data linkage but face constraints due to legislation or the reluctance of collaborators to exchange their sensitive data.

The results of this study benefit MBS providers by revealing that one in nine patients changes institutions for secondary surgery and that these patients tend to be younger with fewer obesity-related diseases. This insight enhances surgeons’ understanding of patient behavior and suggests that their secondary MBS rates, as computed using conventional DATO data, are likely slightly underestimated. The findings also highlight behavioral variability among patients, with younger individuals more commonly represented among patients transferring hospitals. This will only affect hospital performance comparisons if hospital transfers occur more frequently in some hospitals than in others, which seems unlikely. Surgeons may consider modifying their approach to secondary MBS to promote care continuity, given the potential for patients to pursue their desired treatment elsewhere. The study further demonstrates the utility of MPC, and potential collaborations could extend beyond hospitals to include insurers, governmental institutions, or other organizations with complementary data. For instance, obesity management medications (OMMs) are often prescribed by healthcare specialists not affiliated with the MBS institute, and MPC could facilitate analysis of the frequency and impact of (neo-)adjuvant OMM therapy. Beyond MBS, MPC can also be applied in other contexts, for instance, in cancer treatment, where patients often receive neoadjuvant therapy and surgery at different facilities.

The current study has several limitations. Reliance on registry data limited the availability of information on patient motivations, satisfaction, and decision-making, preventing evaluation of the hypothesis that transferring patients may be less satisfied with their outcomes or care. Additionally, outcomes after secondary surgery were not assessed, which would be relevant for determining whether hospital transfer is associated with better patient outcomes. However, this was beyond the study’s scope, as the primary aim was to compare patient and procedure characteristics to assess whether these differed systematically, but would be highly relevant for future research. Furthermore, no gold standard coupling key was available (e.g., social security number), leaving uncertain how many patients could have been linked, and how many patients were missed by coupling on the alternative patient identifier. It seems unlikely that, based on family name, biological sex, and date of birth, patients with certain characteristics would have a higher chance of being captured by the API than others. Therefore, it is not expected that this would have biased the results. Patients who transferred to a hospital abroad will also not be included in the current analysis, but this number is expected to be minimal. The current study also poses several challenges in data interpretation. Although the differences in characteristics between both groups may underlie the likelihood of hospital transfer, the assumption that younger, healthier patients are more likely to be unsatisfied with their treatment remains a hypothesis, and the actual reason for the transfer remains unknown. Additionally, the interval between the surgeries for patients who transferred hospitals was approximately 1 year longer, so the chance of RWG is larger. However, as the BMI of patients transferring hospitals was lower than that of patients who stayed, it does not change the assumption that patients who transferred were more critical of their outcomes. A limitation regarding the MPC software is that it mainly supports descriptive statistics but not regression analysis, which would allow controlling for other variables. In the context of the current study, this means that part of the observed differences may be due to another factor, e.g., the observed lower percentage of hypertension among patients who transferred to another hospital for secondary MBS may be due to these patients also being younger. Still, this does not essentially change the fact that patients who transfer to another hospital for secondary MBS are a selection of patients, reflected by several factors that might be interrelated.

This was the first time that MPC was applied to real-world data, analyzing approximately 100,000 medical records. The current study has helped in understanding whether secondary surgery outcomes based on patients remaining in their initial hospital, as currently done in DATO [44], could be generalizable to the broader MBS population. When future research evaluates the combined impact of primary and secondary MBS, it is critical to understand the potential bias introduced by missing data on patients who transferred hospitals. Since factors associated with weight loss, such as sex, BMI, and diabetes [4, 44], showed no baseline differences between patients who transferred and those who stayed, it suggests that weight loss results are likely representative. Still, future research should more broadly evaluate outcome differences between the two groups, such as complications and long-term outcomes after the secondary surgery. Additionally, it will be of interest to explore whether other factors are associated with hospital transfer, such as socioeconomic status or other characteristics that could be retrieved from third parties.

## Conclusion

Approximately one in nine patients transferred to a different hospital for secondary MBS. These patients were younger, had lower rates of hypertension and GERD, and were more likely to have undergone gastric bypass, particularly RYGB, as their primary procedure. Despite presenting with lower BMI at the time of secondary MBS, they more frequently sought surgery for RWG. This combination suggests that younger patients may be more susceptible to dissatisfaction with their outcomes and more inclined to seek additional treatment elsewhere. The use of MPC facilitated these new insights, highlighting its significant potential for future research collaborations.

## Supplementary Information

Below is the link to the electronic supplementary material.

ESM 1 (DOCX 33.4 KB)

## Data Availability

Sensitive data cannot be publicly available but can be made available by appropriate request to the corresponding author.
