# Supplementary material for: Hospital Transfer Between Primary and Secondary Metabolic Bariatric Surgery in The Netherlands: A Cross-sectional Multi-party Computation Analysis of Frequency and Associated Factors
Source: Obes Surg. 2025 Oct 20;35(12):5069–79. doi: 10.1007/s11695-025-08284-8 (PMC12722452; doi:10.1007/s11695-025-08284-8)
Supplement: Supplementary file 1 — (DOCX 33.4 KB) [file 11695_2025_8284_MOESM1_ESM.docx]

**Supplementary Table 1. Patient and procedure characteristics at the time of the primary surgery for patients included in the sensitivity analysis.**

|  |  | **Same hospital** | **Hospital transfer** | **p-value** |
| --- | --- | --- | --- | --- |
| **N** |  | 1957 | 261 |  |
|  |  |  |  |  |
| **Age at baseline, mean (SD)** |  | 42.5 (11.8) | 37.7 (11.7) | < 0.001 |
| **Sex, n (%)** | *Female* | 1627 (83.1) | 216 (82.8) | 0.88 |
|  |  |  |  |  |
| **BMI at baseline, mean (SD)** |  | 44.1 (7.0) | 44.0 (6.1) | 0.93 |
|  |  |  |  |  |
| **ASA score, n (%)** | *< 3* | 860 (44.4) | 156 (60.7) | < 0.001 |
|  | *3 +* | 1078 (55.6) | 101 (39.3) |  |
|  |  |  |  |  |
| **Diabetes mellitus, n (%)** | *Not present* | 1331 (80.3) | 207 (82.1) | 0.65 |
|  | *Present without medication* | 110 (6.6) | 13 (5.2) |  |
|  | *Present with medication* | 216 (13.0) | 32 (12.7) |  |
|  |  |  |  |  |
| **Hypertension, n (%)** | *Not present* | 1077 (65.0) | 183 (72.6) | < 0.05 |
|  | *Present without medication* | 215 (13.0) | 29 (11.5) |  |
|  | *Present with medication* | 365 (22.0) | 40 (15.9) |  |
|  |  |  |  |  |
| **Dyslipidemia, n (%)** | *Not present* | 1339 (80.8) | 214 (84.9) | 0.25 |
|  | *Present without medication* | 158 (9.5) | 21 (8.3) |  |
|  | *Present with medication* | 160 (9.7) | 17 (6.7) |  |
|  |  |  |  |  |
| **OSAS, n (%)** | *Not present* | 1368 (82.6) | 211 (83.7) | 0.63 |
|  | *Present without medication* | 148 (8.9) | 18 (7.1) |  |
|  | *Present with medication* | 141 (8.5) | 23 (9.1) |  |
|  |  |  |  |  |
| **GERD, n (%)** | *Not present* | 1288 (78.0) | 216 (86.1) | < 0.05 |
|  | *Present without medication* | 197 (11.9) | 19 (7.6) |  |
|  | *Present with medication* | 167 (10.1) | 16 (6.4) |  |
|  |  |  |  |  |
| **Musculoskeletal pain, n (%)** | *Not present* | 845 (51.1) | 150 (59.5) | < 0.05 |
|  | *Present without medication* | 767 (46.3) | 98 (38.9) |  |
|  | *Present with medication* | 43 (2.6) | 4 (1.6) |  |
|  |  |  |  |  |
| **Type of primary surgery, n (%)** | *LAGB* | 53 (2.7) | 22 (8.4) | < 0.001 |
|  | *SG* | 1158 (59.2) | 119 (45.6) |  |
|  | *Bypass* | 703 (35.9) | 118 (45.2) |  |
|  | *Other* | 43 (2.2) | 2 (0.8) |  |
|  |  |  |  |  |
| **Type of primary bypass*, n (%)** | *RYGB* | 375 (53.5) | 108 (91.5) | < 0.001 |
|  | *OAGB* | 232 (33.1) | 6 (5.1) |  |
|  | *Ring augmented RYGB* | 94 (13.4) | 4 (3.4) |  |
|  | *Missing*** | 2 (0.3) | 0 (0.0) |  |

N = number of patients, SD = standard deviation, BMI = body mass index, ASA = American Society of Anesthesiologists, OSAS = obstructive sleep apnea syndrome, GERD = gastro-esophageal reflux disease, LAGB = laparoscopic adjustable gastric banding, SG = sleeve gastrectomy, RYGB = Roux-en-Y gastric bypass, OAGB = one anastomosis gastric bypass, * = only determined for patients receiving gastric bypass, ** = determined on the total number of patients who potentially could have had the variable filled (numbers do not add up to 100%).

**Supplementary Table 2. Patient and procedure characteristics of secondary metabolic bariatric surgery for patients included in the sensitivity analysis.**

|  |  | **Same hospital** | **Hospital transfer** | **p-value*** |
| --- | --- | --- | --- | --- |
| **N** |  | 1957 | 261 |  |
|  |  |  |  |  |
| **Age at revision, mean (SD)** |  | 45.6 (11.7) | 42.0 (11.7) | < 0.001 |
|  |  |  |  |  |
| **BMI at revision, mean (SD)** |  | 43.1 (7.4) | 38.9 (7.2) | < 0.001 |
|  |  |  |  |  |
| **Type of secondary surgery, n (%)** | *LAGB* | 45 (2.3) | 13 (5.0) | < 0.001 |
|  | *SG* | 32 (1.6) | 21 (8.0) |  |
|  | *Bypass* | 1389 (71.0) | 187 (71.6) |  |
|  | *Other* | 491 (25.1) | 40 (15.3) |  |
|  |  |  |  |  |
| **Type of bypass**, n (%)** | *RYGB* | 1159 (83.6) | 146 (78.1) | 0.11 |
|  | *OAGB* | 157 (11.3) | 31 (16.6) |  |
|  | *Ring augmented RYGB* | 70 (5.1) | 10 (5.3) |  |
|  | *Missing***** | 3 (0.2) | 0 (0.0) |  |
|  |  |  |  |  |
| **Type of intervention, n (%)** | *Conversion* | 1184 (74.2) | 86 (58.5) | < 0.001 |
|  | *Undo* | 27 (1.7) | 7 (4.8) |  |
|  | *Revision* | 384 (24.1) | 54 (36.7) |  |
|  | *Missing***** | 362 (18.5) | 114 (43.7) |  |
|  |  |  |  |  |
| **Type of revision***, n (%)** | *Gastro-enterostomy* | 91 (26.8) | 10 (19.2) | 0.12 |
|  | *Entero-enterostomy* | 23 (6.8) | 5 (9.6) |  |
|  | *Adjustment of limb lengths* | 61 (17.9) | 16 (30.8) |  |
|  | *Other* | 165 (48.5) | 21 (40.4) |  |
|  | *Missing***** | 44 (11.5) | 2 (3.8) |  |
|  |  |  |  |  |
| **Reason for intervention, n (%)** | *Primary non-responder* | 42 (2.6) | 4 (2.7) | < 0.001 |
|  | *Recurrent weight gain* | 373 (23.3) | 72 (49.0) |  |
|  | *Comorbidity progression* | 176 (11.0) | 12 (8.2) |  |
|  | *Excessive weight loss* | 52 (3.3) | 3 (2.0) |  |
|  | *GERD* | 163 (10.2) | 7 (4.8) |  |
|  | *Other* | 792 (49.6) | 49 (33.3) |  |
|  | *Missing***** | 324 (16.6) | 114 (43.7) |  |
|  |  |  |  |  |
| **Years until secondary procedure, mean (SD)** |  | 3.0 (2.1) | 4.4 (2.3) | < 0.001 |

N = number of patients, SD = standard deviation, BMI = body mass index, LAGB = laparoscopic adjustable gastric banding, SG = sleeve gastrectomy, RYGB = Roux-en-Y gastric bypass, OAGB = one anastomosis gastric bypass, GERD = gastro-esophageal reflux disease, * = missing category excluded from analyses, ** = only determined for patients receiving gastric bypass, *** = only determined for patients receiving revisional surgery, **** = determined on the total number of patients who potentially could have had the variable filled (numbers do not add up to 100%).

**Supplementary materials**

*Creation of patient identifiers*

For DATO data, a technical identifying variable (standard patient identifier, SPI) is given to each record, which is unique for each patient operated in that hospital. If a patient receives multiple surgeries in that hospital, these surgeries can be linked in DATO to the same patient. However, suppose patients transfer to a different hospital. In that case, this SPI can no longer be used to connect the primary and the secondary surgery as the hospital performing the secondary surgery will give a new SPI to this patient, which is unique for that patient in their hospital, but different from the SPI in the primary hospital. Therefore, alternative coupling variables (alternative patient identifier, API) were created in the current study to explore if surgeries received in different hospitals could be linked based on variables other than the SPI. As using pseudonymized social security numbers for medical research is prohibited, other pseudonymized data should be utilized that may create a unique combination for patients, such as name and residential details. Therefore, 4 APIs were created: [family name + date of birth], [family name + date of birth + sex], [family name + particle of family name + date of birth], and [family name + particle of family name + date of birth + sex]. All family names were transliterated to ensure identical documentation of family names across different hospitals. As patients moving house may explain a substantial part of hospital transfer decisions, residential details were not considered for the API.

*MPC and determination of API*

Hereafter, all data was locally encrypted, fragmented, and uploaded. The API, derived from family name, birth date, and sex, identified a maximum of nine surgeries per patient, consistent with the maximum recorded in DATO based on the SPI. Given that registrars document the year of prior MBS surgery when the primary MBS was performed elsewhere, at least 326 patients were expected to be identifiable by MPC if the API achieved a 100% match rate. The identification of 275 patient matches was considered realistic, as APIs typically yield fewer matches than 100%, especially when matching needs to occur on data from different institutions.^45,46^ Consequently, this API was used as the alternative patient identifier throughout the study.

*Creating the dataset*

All surgeries (n=98,409) were initially grouped by the SPI, retaining patients who underwent both primary and secondary surgery within the specified period in the first dataset. These are the patients who did not transfer to a different hospital for their secondary surgery (because the procedures could be matched on the SPI). A second dataset was then created using the same surgeries but grouped by the API. Surgeries that did not match on the SPI but matched on the API were subsequently added to the first dataset (these are the patients who transferred to another hospital for the secondary procedure), forming the final dataset for analysis. To verify that these patients transferred hospitals, these procedures also needed to show different hospital identifiers (i.e., identifier indicating where the procedure is performed).

References

45 Kim JW, Choi H, Lim H jeung, Oh M, Ahn JJ. Evaluating Linkage Quality of Population-Based Administrative Data for Health Service Research. *J Korean Med Sci* 2024; **39**. doi:10.3346/JKMS.2024.39.E127,.

46 Li B, Quan H, Fong A, Lu M. Assessing record linkage between health care and Vital Statistics databases using deterministic methods. *BMC Health Serv Res* 2006; **6**: 1–10.
